# REBACIN^®^ inhibits E6/E7 oncogenes in clearance of human papillomavirus infection

**DOI:** 10.3389/fonc.2022.1047222

**Published:** 2022-12-06

**Authors:** Shu-Guang Zhou, Dai-Fei Wu, Hui Yao, Wei-Yu Zhang, Feng-Jiao Tian, Guo Chen, Chun-Fa Zhang

**Affiliations:** ^1^ Department of Gynecology, Anhui Medical University Affiliated Maternity and Child Healthcare Hospital, Anhui Province Maternity and Child Healthcare Hospital, Hefei, Anhui, China; ^2^ Division of Molecular Virology, SR Life Sciences Institute, Clarksburg, MD, United States; ^3^ Key Laboratory of Protein Engineering and Drug Development of Hainan, Haikou, China

**Keywords:** REBACIN, human papilloma virus (HPV), E6/E7, clearance, cervical cancer

## Abstract

Previous studies have demonstrated that REBACIN^®^ intervention eliminates persistent high-risk human papillomavirus (hrHPV) infection. The initial establishment and subsequent progression of cervical cancer mainly depends on two major oncogenes, E6/E7, and previous studies have proposed E6/E7 oncogenes as a target for therapeutic drug development. The aim of this study was to investigate *in vitro* and *in vivo* whether REBACIN^®^ inhibits E6/E7 oncogenes for elucidating the mechanism of REBACIN^®^ in the clearance of persistent hrHPV infection. *In vitro*, after REBACIN^®^ treatment, the growth of both Ca Ski and HeLa cervical cancer cells containing the E6/E7 oncogenes was prevented. In line with this finding is that E6/E7 expression was inhibited, which can be counteracted by the co-application of anti-REBACIN^®^ antibody. These studies demonstrated that REBACIN^®^ can effectively inhibit the growth of cervical cancer cells *via* targeting HPV E6/E7 expression. To further verify this finding in clinic, 108 volunteer patients with persistent hrHPV infections were randomly divided into REBACIN^®^, recombinant human interferon alpha-2b (Immunological drug control), or no-treatment blank control groups, received intravaginal administration of REBACIN^®^, interferon or no-treatment every other day for three months, and then followed up for E6/E7 mRNA assay. In REBACIN^®^ group, 68.57% of patients showed complete clearance of HPV E6/E7 mRNA, which was significantly higher compared to 25.00% in the interferon immunological drug control group and 20.00% in blank control group, confirming that REBACIN^®^ is potently efficacious on clearing persistent hrHPV infections *via* inhibition of HPV E6/E7 oncogenes.

**Clinical trial registration:**

http://www.chictr.org.cn/historyversionpuben.aspx?regno=ChiCTR2100045911, identifier ChiCTR2100045911.

## Introduction

Human papilloma virus (HPV) is a highly conserved double-stranded DNA virus (1, 2), which is one of the most common sexually transmitted viruses (3). Although most infections are naturally cleared by the host’s immune system within 1-2 years, about 10% of the infected people become persistent infected (4). Studies have shown that high-risk HPV (hrHPV) persistent infection is the main cause of cervical cancer (5, 6). It is widely known that cervical cancer is the fourth most common cancer among women in the world (7). According to statistics, there were about 570,000 new cases and 311,000 deaths worldwide in 2018 (8). Persistent infection of hrHPV and integration of its DNA into the human genome are the key carcinogenic basis and mechanism of cervical cancer (9). HPV16 and HPV18 are the most common types resulting in about 70-75% of cervical cancer and 40-60% of cervical precancerous lesions in the world (10, 11), while HPV negative people have almost no risk of cervical cancer (12). Studies have shown that HPV E6 and E7 are the two most important oncogenes in the process of HPV induced lesions or carcinogenesis, which are easy to integrate with host genes in the nucleus and then synthesize oncoproteins E6 and E7 (13). It has been proved that their protein products interact with two key factors p53 and pRb of the tumor-associated signaling pathway respectively, leading to the continuous proliferation and carcinogenesis of cervical malignant cells (14–16).

Currently, the conventional treatments for persistent hrHPV infections include cryotherapy, carbon dioxide therapy, laser therapy, loop electrosurgical excision procedure, cold knife conization, et al. (17). These therapies without tissue specificity are invasive in different degrees. Since no anti-HPV drugs have been approved by FDA so far, interferon, a typical immune antiviral medicine, is occasionally used as an alternative drug applied to anti-hrHPV in China (18–21), which plays a critical role in the innate immune response against viral infections by inhibiting virus replication (22). However, the clinical efficacy of interferon on persistent hrHPV infections was controversial (23).

As a recent coming out antiviral biologic, REBACIN^®^ was reported to be high clinical efficacy on the clearance of persistent hrHPV infections (24, 25). The aim of this study is to further investigate the mechanism of REBACIN^®^ in clearing the persistent hrHPV infection.

## Materials and methods

### Cell culture

Ca Ski (ATCC^®^ CRL1550™) is a human epidermoid cervical cancer cell line, which derived from an epidermoid carcinoma of the cervix metastatic to the small bowel mesentery of a 40 year old Caucasian. HeLa (ATCC^®^ CCL2™) is the first immortal human cell line to be grown in culture, which was isolated in 1951 from a cervical carcinoma derived from a 31-year-old patient. 293T (ATCC^®^ CRL-3216™) is a highly transfectable derivative cell line of human embryonic kidney 293 cells. All these cells were purchased from Shanghai Enzyme Research Biotechnology Co., Ltd, cultured in HyClone Dulbecco’s Modified Eagle medium (DMEM)/High glucose containing 10% Fetal Bovine Serum (FBS), 100 units/mL of penicillin and 100 µg/mL of streptomycin, and incubated at 37°C in an atmosphere of 5% CO_2_ (100% relative humidity).

### SDS-PAGE and western blotting

Ca Ski, HeLa or 293T cells were split and approximately 2.5 x 10^5^ cells per well were plated onto 6-well culture plate containing fresh media with or without 50 µg/ml REBACIN^®^, and then the cells were cultured continuously up to 6 days. After every one day’s culture, the total proteins in each wells were extracted with RIPA Lysis and Extraction Buffer containing 1x Halt™ Protease Inhibitor Cocktail an 5 mM EDTA Solution, separated by 10% regular SDS-PAGE, and transferred to nitrocellulose membrane. The membranes were blocked for 1 h with 5% non-fat milk in KPL wash solution (Beijing XMJ Scientific Co., Ltd) and processed with primary monoclonal antibodies (anti-HPV16 E7 and anti-β-Actin, Santa Cruz Biotechnology; anti-HPV18 E6, Merck Millipore) and subsequently with anti-mouse horseradish peroxidase-conjugated secondary antibody (1:500, Santa Cruz Biotechnology, Inc). The membranes were developed using West Femto ECL Substrate (Beijing Solarbio Science&Technology Co., Ltd), and pictures were taken using Amersham Imager 600 (GE Healthcare Life Sciences). The signal of western blotting was quantified using the ImageJ (National Institutes of Health, USA), and the β-actin, a traditional housekeeping protein, was used as an internal control to normalize total protein loading in each lane. Note that the β-Actin and HPV18 E6 was detected respectively to reduce background in **Figure 4C**.

### Cell viability test

Ca Ski, HeLa or 293T cells were split and approximately 5 x 10^4^ cells per well were plated onto 24-well culture plate containing fresh media with or without 50 µg/ml REBACIN^®^ in the presence or absence of anti-REBACIN^®^ antibody, and then the cells were cultured continuously up to 6 days. After every one day’s culture, the cells in each well were split and stained with Trypan Blue Solution, and the living cells were counted according to the manufacturer’s protocol (Gibco).

### Participants

This clinical observation was approved by the Institutional Ethics Committee of Anhui Province Maternity and Child Healthcare Hospital. Between June 2019 and December 2020, participants with persistent infections of high-risk HPV (hrHPV) for at least twelve months, but without a high level of cervical lesions [no more than grade one cervical intraepithelial neoplasia (CIN 1) according to the examination of colposcopy and biopsy] were enrolled in the Department of Gynecology at Anhui Province Maternity and Child Healthcare Hospital.

Inclusion criteria of participants: (1) women between 18 and 70 years old with sexual life, but non-pregnant or lactating; (2) persistent hrHPV infection for more than twelve months; (3) without a high level of cervical lesions [no more than grade one cervical intraepithelial neoplasia (CIN 1) according to the examination of colposcopy and biopsy]; (4) no previous anti-HPV intervention. Exclusion criteria of participants: (1) with a history of allergy, smoking, oral contraceptive and HPV vaccination; (2) with severe heart, lung, liver and kidney dysfunction; (3) women who can’t complete the designed drug administration due to menstrual disorder. There was no significant difference (p>0.05) in the age, E6/E7 mRNA expression level, cytological abnormality of the participants among REBACIN^®^, recombinant human interferon alpha-2b and no-treatment blank control group (Data not shown).

### Study design

After understanding details of the investigation and agreeing to comply with the research process, patients signed a written informed consent form and then participated in this clinical observation. Eligible volunteers were screened out according to the inclusion and exclusion criteria. A random digital table was generated *via* a computer randomized numbering system. According to the digital table, eligible patients were randomly assigned into experimental groups.

All eligible participants received intravaginal administration of either REBACIN^®^ (0.5 g compounding agent per dose, the Key Laboratory of Protein Engineering and Drug Development of Hainan, Haikou, China) or recombinant human interferon alpha-2b (100,000 IU per suppository, Anhui Anke Bioengineering (Group) Co., Ltd., Anhui, China) every other day for three months, except during the menstrual period. The patients were then followed-up during four months after their drug withdrawal. The expression level of hrHPV E6/E7 mRNA was detected using HPV E6/E7 mRNA assay as shown in the following.

### HPV E6/E7 mRNA assay by QuantiVirus

HPV E6/E7 mRNA analysis were carried out according to the manufacturer’s instructions of QuantiVirus HPV E6/E7 mRNA assay (Kodia Biotechnology, China), which detects HPV oncogenes E6/E7 mRNA of 14 high-risk HPV subtypes (16, 18, 31, 33, 35, 39, 45, 51, 52, 56, 58, 59, 66 and 68). This test utilizes branched DNA (bDNA) to capture the target mRNA without either mRNA purification or reverse transcription−polymerase chain reaction. The E6/E7 mRNAs were quantified as relative luminescence units (RLU) by the KODIA QuantiViurs Luminometer. If RLU was more then 1.0, the patient was considered as positive of high-risk HPV E6/E7 mRNAs in the assay (26). Otherwise, the patient was negative.

### Cervical cytology

According to routine method, thinPrep cytologic test (TCT) was used to identify abnormal cells at the squamocolumnar junction, where cervical dysplasia and cancers mostly develop. The observations were reported according to the Bethesda 2014 guidelines (27).

### Statistical analysis

According to significance level (α) and power (1-β) requirements of the clinical observation, the number of cases for the REBACIN^®^ group, interferon alpha-2b group and no-treatment blank control group was determined in a ratio of 1:1:1. The evaluation indicators are qualitative indicators. According to the efficacy of pre-test and statistical requirements (α=0.05, and β=10%), the minimum sample size required for this clinical observation was obtained.

The data from cell viability test and western blotting were analyzed by Repeated Measures ANOVA followed by Bonferroni test (GraphPad Prism 6). The counting data was analyzed *via* the chi-square test. All the p-values reported were two-sided, and p-values < 0.05 indicated statistical significance.

## Results

### REBACIN^®^ inhibits cell growth of cervical cancer cell line Ca Ski

**Figure 1:**
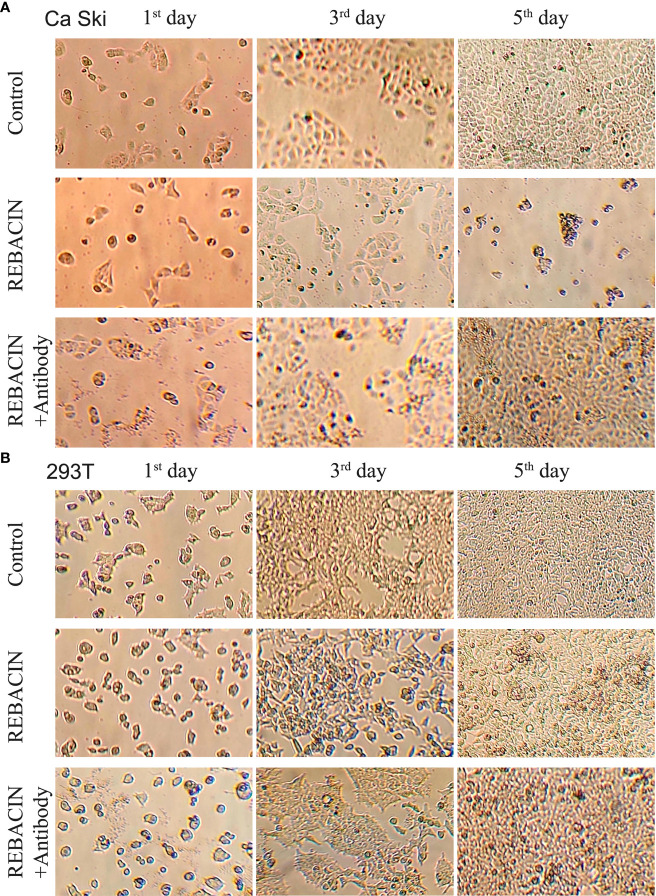
Effect of REBACIN^®^ on the growth of different cell lines. Ca Ski **(A)** or 293T **(B)** cells in 24-well plate were treated or not treated by 50 μg/ml REBACIN^®^ or REBACIN^®^/anti-REBACIN^®^ antibody for up to 6 days, and the representative images in the 1^st^, 3^rd^, and 5^th^ day were shown.

**Figure 2:**
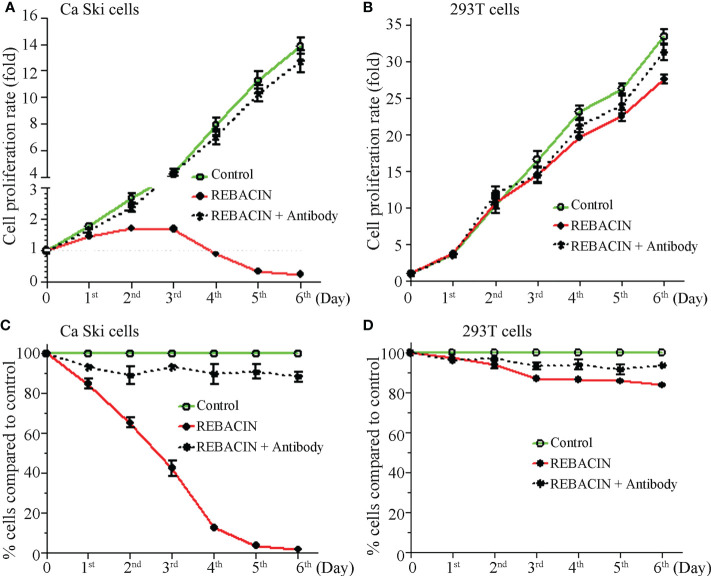
Effect of REBACIN^®^ on the growth of different cell lines. Ca Ski **(A, C)** or 293T **(B, D)** cells in 24-well plate were treated or not treated by 50 μg/ml REBACIN^®^ or REBACIN^®^/anti-REBACIN^®^ antibody for up to 6 days, and the total amount of alive cells in each well was counted every 24 hours. During each count, the cell proliferation rate at the beginning stage was set as 1 **(A, B)**; the total alive cells in each well without any treatment (control) were set as 100%, and the total alive cells in treated well were normalized to its control **(C, D)**. Data show mean ± SEM of seven independent experiments. REBACIN^®^ versus control: p < 0.05 in **(A)** and p < 0.01 in **(C)**.

HPV E6/E7 oncoproteins are thought to immortalize cells and maintenance of HPV-associated cancer, primarily through interference with the p53 and pRB tumor suppressor proteins (28–30). We therefore investigated whether REBACIN^®^ treatment affects the growth of a human epidermoid cervical cancer cell line of Ca Ski cells, which stably express HPV16 E6/E7 oncoproteins. As shown in **Figure 1A**, the treatment of REBACIN^®^ did significantly inhibit the growth of Ca Ski cells. Five days after the treatment, there were only few cells alive compared to the confluence cells in control without the treatment of REBACIN^®^, and this growth inhibition can be almost completely counteracted under co-application of anti-REBACIN^®^ antibody. Otherwise, in a cancer cell line of 293T derived from human embryonic kidney 293 epidermoid cells without containing HPV E6/E7 genes, REBACIN^®^ almost has no influence in 293T cell growth (**Figure 1B**). In quantitative analysis, six days after the culture, the total cells of Ca Ski proliferated almost 14 folds under normal growth condition whereas alive cells decreased around 75% in the presence of REBACIN^®^, which the growth inhibition in the presence of REBACIN^®^ could be blocked with co-application of anti-REBACIN^®^ antibody (**Figure 2A**). In other words, the growth of Ca Ski cells was inhibited up to 98% compared to the control (**Figure 2C**). For the cancer cells of 293T without expression of the E6/E7 oncoproteins, REBACIN^®^ only slightly influenced cell growth, and anti-REBACIN^®^ antibody didn’t significantly counteract this slight inhibition (**Figures 2B, D**).

### REBACIN^®^ inhibits the expression of HPV16 E7 oncoprotein

**Figure 3:**
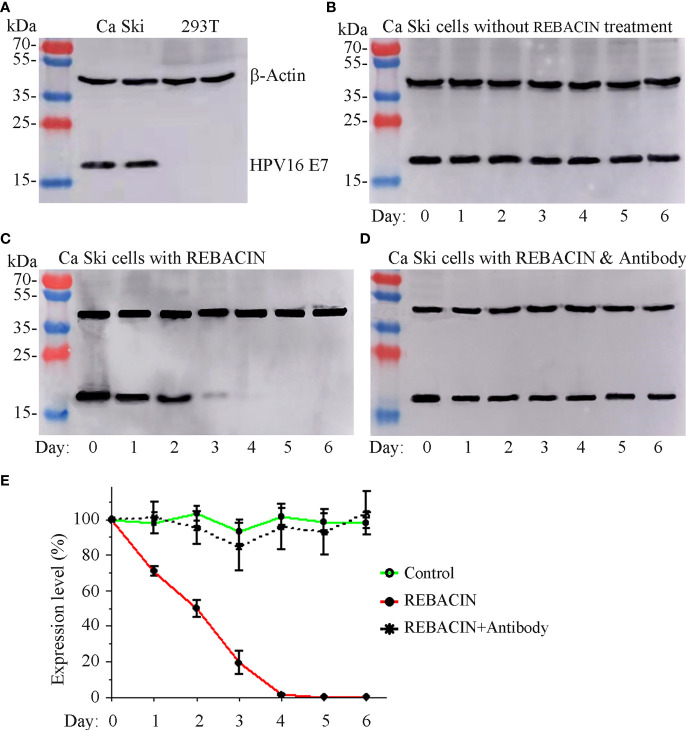
Effect of REBACIN^®^ on the expression of HPV16 E7. Ca Ski or 293T cells in 6-well plate were treated or not treated by 50 μg/ml REBACIN^®^ for up to 6 days. The expression of β-actin and HPV16 E7 in different cell lines was detected by western-blot in **(A)**, and their expression levels in each well every 24 hours under different conditions were shown in **(B–D)**, respectively. Representative images were shown in **(A–D)**, and the relative quantitative data from **(B–D)** were analyzed and shown in **(E)**, which the expression level at 0 day was set as 100%. Data show mean ± SEM of three independent experiments. REBACIN^®^ versus control: p < 0.001 in **(E)**.

Since REBACIN^®^ influenced the growth of Ca Ski cells with expression of HPV16 E6/E7 oncoproteins, and only had slight influence in the growth of 293T cells without expression of HPV16 E6/E7 oncoproteins. We therefore investigated whether REBACIN^®^-mediated inhibition of Ca Ski’s growth is *via* the inhibition of E6/E7 oncoproteins. As expected, HPV16 oncoprotein E7 was detected in Ca Ski cells, and was under detectable in 293T cells (**Figure 3A**). The expression of HPV16 E7 oncoprotein was relative stable during six-day culture (**Figure 3B**). However, in the presence of REBACIN^®^, the expression of HPV16 E7 oncoprotein was gradually decreased as the culture time increased. Five days after the treatment, oncoprotein E7 was under detectable in Ca Ski cells (**Figures 3C, E**). The REBACIN^®^-mediated inhibition of HPV16 E7 expression could be counteracted under co-application of anti-REBACIN^®^ antibody (**Figures 3D, E**).

### REBACIN^®^ influences the growth of HeLa cells

**Figure 4:**
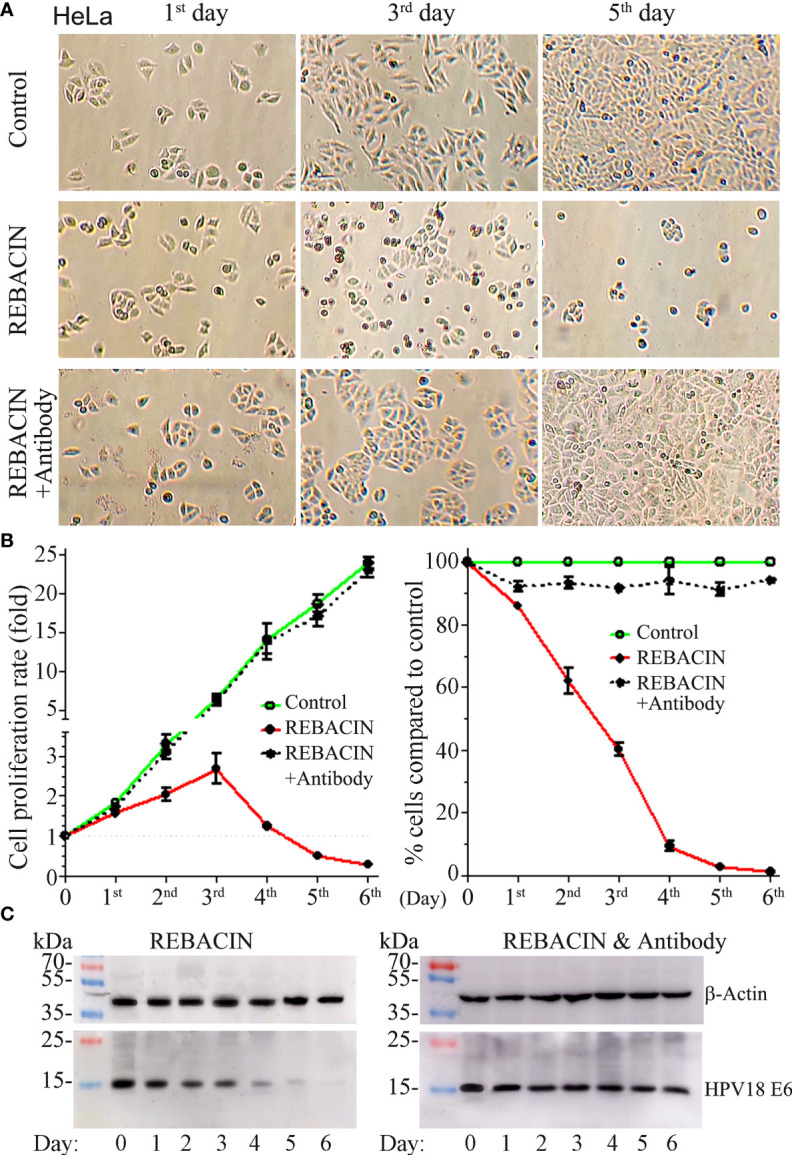
REBACIN^®^ inhibits HeLa cell’s growth *via* the suppression of HPV18 E6. HeLa cells in 24-well plate were treated or not treated by 50 μg/ml REBACIN^®^ or REBACIN^®^/anti-REBACIN^®^ antibody for up to 6 days, and representative images in the 1^st^, 3^rd^, and 5^th^ day were shown in **(A)**. The total amount of alive cells in each well was counted every 24 hours, and their quantitative data show mean ± SEM of four independent experiments. REBACIN^®^ versus control: p < 0.05 in **(B)**. The expression level of β-actin and HPV18 E6 was detected by western-blot, and representative images were showed in **(C)**.

HeLa is a cervical cancer cell line, and have been reported to contain human papilloma virus 18 (HPV18) sequences. As expected, the treatment of REBACIN^®^ did influence this cervical cancer cell’s growth. As shown in **Figure 4A**, REBACIN^®^ treatment markedly inhibited the cell growth, which exhibited a time-dependent manner, and this inhibition can be blocked in the presence of anti-REBACIN^®^ antibody (**Figures 4A, B**). In line with this find is that the expression level of HPV18 E6 oncoprotein was impressively decreased after the REBACIN^®^ treatment, and this effect can be blocked by the addition of anti-REBACIN^®^ antibody (**Figure 4C**), indicating that REBACIN^®^ inhibits HeLa cell’s growth *via* the suppression of HPV18 E6 oncoprotein.

### Strong effect of REBACIN^®^ on hrHPV E6/E7 mRNA suppression

*In vitro* study in cervical cancer cells demonstrated that REBACIN^®^ inhibited cell growth *via* inhibition of HPV E6/E7 oncoproteins, we thus further investigated in clinical observation whether REBACIN^®^ affects the expression level of hrHPV E6/E7 mRNA. In this study, the participants were randomly divided into REBACIN^®^, interferon or no-treatment blank control groups, and were treated intravaginally by REBACIN^®^, recombinant human interferon alpha-2b or no-treatment every other day for three months, except their menstrual period. All patients were then followed up for a test of hrHPV E6/E7 mRNA during four months after the intervention, and the effect of REBACIN^®^ on the expression of hrHPV E6/E7 mRNA were evaluated. Of the 108 participants, 106 (98.15%) patients had all information available for statistical analysis. As shown in **Table 1**, 68.57% (24/35) of patients were tested as negative for hrHPV E6/E7 mRNA after three-month administration in REBACIN^®^ treatment group, which was significantly higher in comparison with 20.00% (7/35) in the blank control group and also 25.00% (9/36) in the interferon alpha-2b control group. This finding demonstrates that REBACIN^®^ markedly inhibits the expression of hrHPV E6/E7 oncogenes.

**Table 1:** Efficacy of REBACIN^®^ on the clearance of hrHPV E6/E7 mRNA.

Group	Total Case	Clearance Rate
REBACIN^®^	35	68.57% (24/35) ^***^
Interferon	36	25.00% (9/36) ^*^
Blank Control	35	20.00% (7/35)

Note that: *P<0.05, and ***P<0.001 versus blank control analyzed by the Chi-square test (χ^2^).

### No significant side effect was observed in this study

Clinical observation has shown that, for all participants, there was no significant side effect during and after the intervention in this clinical observation. Moreover, routine physical examinations in all participants before and after intervention indicated that the participant’s blood routine, liver function (ALT and AST), kidney function (BUN and Cr) and other tests were in the normal range. However, REBACIN^®^ or interferon alpha-2b induced mild vaginal itch (five patients) was occasionally observed. No other side effects were reported so far.

## Discussion

Although the incidence of cervical cancer has dropped in industrialized countries over the past decade, the rate of decline has been much slower and even increasing in some of developing countries. In developing countries, the cervical cancer accounts for about 12% of all female cancers, and up to 90% mortality rate occurs disproportionately (31). The number of patients diagnosed with persistent hrHPV infections in hospitals has been kept high in China during recent several years, becoming a high-risk population of cervical cancer. The standard clinical therapy of serious hrHPV infection and hrHPV-induced cervical intraepithelial lesion is surgery, which could lead to potential adverse effects including future fertility (32–34). However, there are currently no available FDA-approved antiviral drugs targeting HPV infections (35).

REBACIN^®^ was recently reported to be efficacious on the clearance of persistent hrHPV infection (24, 25), and this innovative non-invasive clinical intervention creates a new clinical approach and option in addition to standard invasive treatment of surgery for the patients with persistent hrHPV infection and its related diseases. In particular, patients, especially in the developing country, can use it by themselves under the guidance of doctors without hospitalization.

On the basis of previous research (24, 25), this study attempts to further confirm and verify that REBACIN^®^ eliminates persistent high-risk HPV infection *via* inhibition of hrHPV E6 and E7 oncogenes. We found *in vitro* that REBACIN^®^ inhibits and even blocks the growth of human cervical cancer cell lines of both Ca Ski and HeLa by inhibition of HPV E6/E7 expression. Moreover, in a clinical observation, three-month treatment of REBACIN^®^ resulted in 68.57% of the patients with a completed suppression of hrHPV E6/E7 mRNA in REBACIN^®^ treatment group, while 25.00% in the interferon alpha-2b control group (a typical immunological drug), and 20.00% in the no-treatment blank control group. This finding strongly supports that the effect of REBACIN^®^ on the clearance of hrHPV infection is *via* inhibition of hrHPV E6/E7 expression.

High-risk HPV E6/E7 oncogenes play a key role not only in cervical intraepithelial lesion and the development of cervical cancer (36), but also in viral replication and maintenance (37, 38). HPV E6/E7 RNA interference was suggested as a promising therapeutic candidate for a wide variety of HPV-associated cancers (28–30). Several strategies targeted at HPV E6/E7 have been investigated for the development of therapeutic vaccines (39). However, all these strategies are still under investigation. A certain level of E6/E7 expression is required for HPV16 replication and maintenance in the host cells (38). If the expression of the E6/E7 oncogenes is completely suppressed, the viral replication and maintenance will not continue and the hrHPV persistent infection will be eliminated. Thus, this study demonstrated REBACIN^®^ as a non-invasive intervention therapy with high clinical efficacy in the clearance of persistent high-risk HPV infection *via* inhibition of hrHPV E6/E7 expression. As for how REBACIN^®^ inhibits the expression of E6/E7 oncogenes, further investigation is needed.

## Data availability statement

The original data presented in the study are included in the article material. Further inquiries can be directed to the corresponding authors.

## Ethics statement

The studies involving human participants were reviewed and approved by The Institutional Ethics Committee of Anhui Province Maternity and Child Healthcare Hospital. The patients/participants provided their written informed consent to participate in this study.

## Author contributions

C-FZ and GC conceived and designed the study. S-GZ, D-FW, HY, W-YZ and F-JT performed the experiments and carried out the data collection. S-GZ and D-FW analyzed the data and drafted the original manuscript. C-FZ and GC edited the manuscript. All authors contributed to the article and approved the submitted version.
